# Correlation Between SARS-Cov-2 Vaccination, COVID-19 Incidence and Mortality: Tracking the Effect of Vaccination on Population Protection in Real Time

**DOI:** 10.3389/fgene.2021.679485

**Published:** 2021-06-02

**Authors:** Kiyoshi F. Fukutani, Mauricio L. Barreto, Bruno B. Andrade, Artur T. L. Queiroz

**Affiliations:** ^1^KAB Group, Goncalo Moniz Institute, Oswaldo Cruz Foundation, Salvador, Brazil; ^2^Multinational Organization Network Sponsoring Translational and Epidemiological Research Initiative, Salvador, Brazil; ^3^Center of Data and Knowledge Integration for Health, Oswaldo Cruz Foundation, Salvador, Brazil

**Keywords:** COVID19, vaccine, worldwide, epidemiology, virosis

## Abstract

Coronavirus disease 19 (COVID-19) has struck the world since the ending of 2019. Tools for pandemic control were scarce, limited only to social distance and face mask usage. Today, upto 12 vaccines were approved and the rapid development raises questions about the vaccine efficiency. We accessed the public database provided by each country and the number of death, active cases, and tests in order to evaluate how the vaccine is influencing the COVID-19 pandemic. We observed distinct profiles across the countries and it was related to the vaccination start date and we are proposing a new way to manage the vaccination.

## Introduction

A new SARS-Cov-2 associated disease is commonly known as coronavirus disease 19 (COVID-19) and present as a spectrum of clinical manifestations ranging from asymptomatic, minor flu-like symptoms to acute respiratory distress syndrome, pneumonia, and death (Sharma et al., 2020). Rapidly, the COVID-19 became a worldwide public health emergency and several attempts to control its dissemination were proposed by non- pharmacological interventions. The most used interventions were social distancing and the use of face masks, since there was no antiviral treatment or any effective vaccine (Randolph and Barreiro, 2020). In the last year, several vaccine candidates were in development, as a result of the great effort to contain the pandemic. However, due to the rapid vaccine development, uncertain questions have been raised in common media, such as the vaccine production capacity to attempt the global demand and its efficacy (Chen, 2020). The emergencial development of COVID-19 vaccines occurred extremely fast, integrating various tools and vaccine platforms. In the future, this technology will be useful to quickly develop vaccines against other new emerging diseases (Hodgson, 2020). Each government must have its own platform for vaccination tracking, in order to perform the monitoring of vaccine coverage and to early identification of possible adverse effects (Hanney et al., 2020). In 2020, we developed a recursive sub-typing screening surveillance system able to perform automated genomic surveillance accessing all the sequences deposited in different repositories for mining, subtyping and performing a genomic surveillance. This system was also able to evaluate the vaccination profile in Brazil by accessing the global vaccination program dataset. As a result the system was able to identify new zika lineage occurrences (Kasprzykowski et al., 2020) and revealed a decrease in children vaccination in the last years in Brazil (Césare et al., 2020). Given the relevance of the SARS-Cov-2 pandemic, we adapted our system to track the association between implementation of vaccines, occurrence of new cases and mortality over time.

## Materials and Methods

To evaluate the COVID-19 vaccination, we developed an application of this tool to real-time access a public access COVID-19 database provided in a cross-country database of COVID-19 (Hasell et al., 2020). CaVaCo (Cases, Vaccinations, and COVID-19) tool allows us to retrieve the COVID-19 cases, deaths and vaccination data to compare and correlate countries vaccination coverage with other parameters. The tool was developed in R (Wickham and Grolemund, 2016), powered to download and standardize the data automatically. As a result the correlation between number of daily vaccines by number of new cases, number of new deaths and number of tests is performed, using the spearman correlation. To access the real-time tool, access: http://kaiju.bahia.fiocruz.br/sample-apps/CaVaCo/.

## Perspective

**FIGURE 1:**
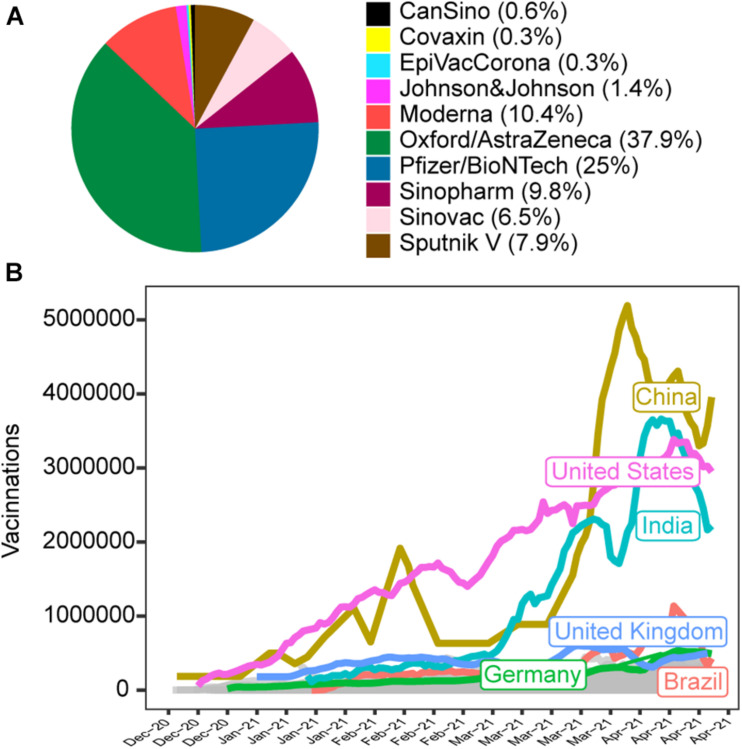
Worldwide distribution of vaccines. **(A)** Proportional of usage of vaccines by countries represented in a sector graph. **(B)** Daily distribution in all countries with a top six countries highlighted.

So far (April 23, 2021), there are 10 vaccines approved and being used worldwide (until: CanSino, Covaxin, EpiVacCorona, Johnson & Johnson, Moderna, Oxford/AstraZeneca, Pfizer/BioNTech, Sinopharm, Sinovac, and Sputnik V). From the 193 countries that started vaccination (List of countries below) the majority have started the vaccination program using Oxford/AstraZeneca vaccine (*n* = 135, 37.9%) while 25% had chosen the Pfizer/BioNTech and 10.4% Moderna and the remaining 26.7% used CanSino, Covaxin, EpiVacCorona, Johnson & Johnson, Sinopharm, Sinovac, and Sputnik V (Figure 1). Using the date available up to April 23, 2021, we performed a correlation analysis between the numbers of new cases with the daily vaccinations. As a result, 60 countries presented positive correlations (Table 1) and 27 countries with negative correlation (Table 1). Despite the vaccination, the number of new cases has been still increasing in these countries. This finding reinforces the need to keep social distance and the use of face masks recommendations to reduce the virus transmission. In other hand the decreasing number of vaccinations and cases can depict a positive correlation and the number of days and the percent of vaccine population could inform how successfully the vaccination program is going. These recommendations should be employed until at least the immunization starts to show a significant reduction in the number of cases (Ahmed et al., 2021). The countries with negative correlation started to have a reduction in the number of new cases and the vaccination should maintain the decreasing number of cases, since the isolation alone is not able to control the COVID-19 (Hellewell et al., 2020). The same approach has employed with the number of new deaths and we observed 37 countries with positive correlations and 33 countries have negative correlations (Table 1). These results show that implementation of vaccines is not the final solution and the maintenance of the non-pharmacological interventions should not be abandoned once the increase of new cases and deaths are indicating the population remains vulnerable to SARS- COV2 infection (Billon-Denis and Tournier, 2020). On the other hand, the negative correlation in certain countries point to a success en route to the vaccination program in reducing both the COVID-19 cases and related deaths. Only 5 countries have positive correlation between the number of vaccination and the number of tests positive for COVID-19 in February 2, 2021 (This data was discontinued). These countries remained testing the population even though the vaccination started. Only Sweden presented a negative correlation (Supplementary Table 1). This approach is useful for pandemic surveillance and the stop of population testing is dangerous and does not prevent the identification of new waves (Holt, 2021). The correlation between the cases/deaths and the vaccination numbers could be a powerful indicator of disease control, since a certain coverage is required for population protection. The continuous follow up of the correlation patterns from the beginning of the vaccination can be used to track the immunization program in each country. Additionally with the genomic surveillance can reveal how the vaccine responds against the introduction of new COVID-19 variants, as previously described (Korber et al., 2020). The present study has some limitations, such as the heterogeneity of strategies applied by the different countries indicated that an individual analysis of specific countries should be performed to evaluate in more granularities the distinct epidemiologic situations, to minimize this effect the number of days used in the correlation analysis are depicted in the table. Some countries displayed substantial missing data or discontinue measuring few variables, like the number of test to COVID-19 in their database. This analysis uses numerical measurements and it cannot reflect the entire national behavior or public politics. Also the present analysis cannot handle or correct numeric bias or outlier interferences. However, taking together these data and applying statistics methods allowed us to monitor the vaccination process in countries or in sub national units. Recursive evaluation of immunization and COVID-19 morbimortality has potential to provide a unique tool to aid decision-making strategies to overcome the current pandemic.

**TABLE 1:** Correlation between the numbers of vaccines against the number of new cases and new deaths in the country have started the vaccination.

	Cases		Deaths		N_of_days
	Rho coefficient	*P*-value	Rho coefficient	*P*-value	
Afghanistan	0.737	9.67876E-11	0.297	0.026230439	56
Albania	–0.702	1.5955E-15	–0.132	0.200294507	96
Algeria	–0.355	0.124947226	–0.023	0.923070238	20
Andorra	0.171	0.127808994	–0.123	0.2738076	81
Angola	0.762	2.04949E-10	0.074	0.610931829	49
Antigua and Barbuda	0.596	3.99133E-05	0.247	0.119180346	41
Argentina	0.274	0.003181829	0.035	0.71201508	114
Australia	0.472	0.000138566	0.139	0.289253571	60
Austria	0.581	8.1842E-12	–0.413	4.09028E-06	116
Azerbaijan	0.665	2.0045E-13	0.428	1.52276E-05	95
Bahamas	–0.084	0.817442415	0.432	0.213058411	10
Bahrain	0.821	1.53122E-30	0.562	2.29498E-11	120
Bangladesh	–0.129	0.235845636	–0.098	0.369853974	86
Barbados	0.769	1.76922E-13	0.249	0.049489179	63
Belarus	–0.581	2.97413E-09	–0.495	9.68988E-07	88
Belgium	0.553	1.765E-10	–0.339	0.000228344	114
Belize	–0.152	0.422511874	–0.247	0.187607437	30
Bolivia	–0.131	0.239039865	–0.47	7.18512E-06	83
Botswana	–0.738	0.262135213	–0.632	0.367544468	4
Brazil	0.306	0.002733153	0.641	3.29746E-12	94
Bulgaria	0.602	2.1644E-12	0.376	4.43204E-05	112
Cambodia	0.645	2.88954E-05	0.407	0.015217248	35
Canada	0.111	0.211429123	–0.676	1.46437E-18	129
Chile	0.521	1.6939E-09	0.267	0.003674764	117
China	–0.548	2.11955E-11	–0.363	2.52497E-05	128
Colombia	0.763	3.73228E-13	0.53	8.10472E-06	63
Costa Rica	–0.005	0.968928062	–0.133	0.302256683	62
Cote d’Ivoire	–0.667	1.21673E-07	–0.092	0.52432257	50
Croatia	0.422	3.31679E-06	–0.2	0.033482549	113
Cyprus	0.592	4.55219E-11	–0.309	0.001502789	103
Czechia	–0.276	0.002743769	–0.03	0.745564613	116
Denmark	–0.156	0.096400077	–0.711	5.64755E-19	115
Dominican Republic	–0.197	0.139137365	–0.214	0.106540076	58
Ecuador	0.17	0.111648671	0.125	0.24157091	89
Egypt	0.334	0.007902683	–0.343	0.006353965	62
El Salvador	0.065	0.658542705	–0.45	0.001318082	48
Equatorial Guinea	0.411	0.209233119	0.181	0.594070448	11
Estonia	0.403	7.97801E-06	0.528	1.37048E-09	115
Eswatini	0.05	0.839790752	0.012	0.961032401	19
Finland	0.496	3.53742E-08	–0.002	0.984810853	110
France	0.419	3.22325E-06	–0.097	0.302673079	115
Gabon	0.017	0.964546145	0.152	0.696613433	9
Gambia	–0.363	0.183775848	–0.038	0.891645336	15
Georgia	0.714	3.3297E-07	0.298	0.06509552	39
Germany	0.077	0.409459266	–0.672	1.45921E-16	116
Ghana	0.536	0.0027202	0.586	0.00083082	29
Greece	0.807	1.20134E-27	0.564	5.35719E-11	115
Guatemala	0.162	0.24210615	0.089	0.521713212	54
Guinea	0.354	0.14947453	0.323	0.191635298	18
Guyana	0.593	3.10376E-07	0.463	0.000131385	63
Honduras	0.22	0.184078772	0.133	0.427631584	38
Hungary	0.719	1.5585E-19	0.742	2.16927E-21	115
India	0.881	1.2858E-32	0.681	1.60918E-14	97
Indonesia	–0.874	1.8398E-32	–0.721	2.69614E-17	100
Iran	0.501	6.28703E-06	0.578	8.77196E-08	73
Iraq	0.756	1.25194E-09	0.52	0.00021189	46
Ireland	–0.801	8.60646E-26	–0.379	4.51725E-05	110
Israel	0.711	4.70919E-20	0.696	5.44762E-19	122
Italy	0.349	0.000123357	–0.146	0.117036098	116
Jamaica	0.132	0.449153159	0.101	0.563670349	35
Japan	0.729	8.62366E-12	–0.583	4.23837E-07	64
Jordan	0.578	5.89729E-10	0.819	1.29465E-24	97
Kazakhstan	0.788	4.2308E-18	0.258	0.020992955	80
Kenya	0.152	0.302319625	0.645	7.30516E-07	48
Kuwait	0.92	5.90966E-38	0.825	9.00643E-24	91
Kyrgyzstan	0.364	0.200594765	0.444	0.111919261	14
Latvia	–0.334	0.000993721	–0.383	0.000139038	94
Lebanon	–0.176	0.151851246	–0.394	0.000887308	68
Liechtenstein	–0.027	0.799263967	–0.209	0.044703081	93
Lithuania	–0.24	0.009513178	–0.78	5.82682E-25	116
Luxembourg	0.384	0.000573533	–0.127	0.270387032	77
Malawi	0.057	0.744125998	–0.091	0.604507315	35
Malaysia	–0.321	0.015926702	–0.189	0.163141237	56
Maldives	–0.25	0.027255853	–0.146	0.20234993	78
Mali	0.382	0.198295213	0.377	0.203554459	13
Malta	–0.423	2.18047E-05	–0.164	0.113838248	94
Mauritania	0.328	0.274642718	0	1	13
Mexico	–0.6	7.14745E-13	–0.388	1.41634E-05	118
Moldova	–0.482	0.000520045	–0.075	0.614125617	48
Monaco	0.057	0.562869113	0.177	0.072673617	104
Mongolia	0.596	4.87635E-06	0.312	0.027613831	50
Montenegro	–0.83	1.39318E-16	–0.002	0.984983613	61
Morocco	–0.405	0.000130867	–0.151	0.171129017	84
Mozambique	0.414	0.125247712	0.468	0.078739952	15
Myanmar	0.534	2.23273E-05	0.617	4.17262E-07	56
Namibia	–0.233	0.199540931	–0.07	0.703377868	32
Nepal	–0.536	1.24587E-07	–0.208	0.056620063	85
Netherlands	0.563	3.62332E-09	–0.578	1.09621E-09	94
Nigeria	–0.47	0.000658627	–0.323	0.023420163	49
North Macedonia	–0.184	0.141337842	0.308	0.012558504	65
Norway	0.194	0.037338479	–0.193	0.0391789	115
Oman	0.744	1.70361E-15	0.727	1.54321E-14	81
Pakistan	0.85	1.10786E-16	0.275	0.039973983	56
Palestine	0.375	0.078301362	0.018	0.933625998	23
Panama	–0.607	1.15805E-10	–0.655	1.03082E-12	93
Papua New Guinea	–0.134	0.694830743	–0.304	0.364208929	11
Paraguay	0.532	2.39756E-05	0.735	1.10315E-10	56
Peru	0.152	0.26207624	0.087	0.522670769	56
Philippines	0.743	2.96125E-10	0.508	0.000120473	52
Poland	0.584	7.38759E-12	0.219	0.018833951	115
Portugal	–0.785	1.84683E-25	–0.801	3.53171E-27	116
Qatar	0.953	1.57075E-63	0.76	5.05664E-24	121
Romania	0.295	0.001391266	0.488	3.06984E-08	115
Russia	–0.942	1.35517E-61	–0.731	1.17779E-22	128
Rwanda	–0.183	0.16240758	0.152	0.247662124	60
Saint Lucia	0.09	0.540947937	–0.121	0.41287011	48
Saint Vincent and the Grenadines	0.201	0.336375569	0.261	0.207778613	25
San Marino	–0.599	3.45821E-05	–0.096	0.54909611	41
Sao Tome and Principe	–0.154	0.600169081	–0.379	0.182026033	14
Saudi Arabia	0.718	4.51089E-18	0.767	8.35156E-22	106
Senegal	–0.218	0.096446802	–0.153	0.245958698	59
Serbia	0.19	0.057375795	–0.016	0.87312687	101
Seychelles	0.011	0.921462236	0.113	0.324517823	78
Singapore	–0.107	0.298611556	0.044	0.670464492	97
Slovakia	–0.489	7.71715E-08	–0.127	0.189701311	108
Slovenia	–0.323	0.000406873	–0.803	2.20051E-27	116
South Africa	0.049	0.717938189	0.019	0.891011593	56
South Korea	0.506	7.03271E-05	0.033	0.808115573	56
Spain	–0.663	1.22683E-10	–0.644	5.86039E-10	74
Sri Lanka	0.151	0.203361052	0.089	0.452643727	73
Suriname	–0.254	0.056169252	–0.25	0.060694914	57
Sweden	0.222	0.080308246	–0.565	1.38924E-06	63
Switzerland	0.338	0.008830615	–0.604	4.1967E-07	59
Taiwan	0.018	0.927252954	–0.203	0.290198758	29
Thailand	0.531	0.000104799	–0.034	0.819580758	48
Togo	–0.299	0.09095591	–0.03	0.869058406	33
Trinidad and Tobago	0.393	0.002273168	0.239	0.071135107	58
Tunisia	0.829	3.90157E-11	0.796	8.3898E-10	40
Turkey	0.383	9.18204E-05	–0.005	0.96031409	99
Uganda	0.369	0.022487201	0.11	0.509511091	38
Ukraine	0.679	6.5518E-09	0.669	1.2839E-08	57
United Arab Emirates	0.311	0.001169005	0.035	0.722547459	106
United Kingdom	–0.557	3.83837E-10	–0.521	7.28931E-09	108
United States	–0.78	1.94261E-26	–0.774	8.82158E-26	123
Uruguay	0.799	7.33099E-13	0.886	1.18631E-18	53
Uzbekistan	0.815	1.19672E-05	0.124	0.60357372	20
Venezuela	0.484	0.000278054	0.558	1.74225E-05	52
Zambia	–0.6	0.208	–0.676	0.140357387	6
Zimbabwe	0.056	0.664808883	–0.269	0.034558192	62

*Countries without enough number of observations to perform a correlation analysis were excluded The up to date table is available in http://kaiju.bahia.fiocruz.br/sample-apps/CaVaCo/ List of countries: Afghanistan, Albania, Algeria, Andorra, Angola, Anguilla, Antigua and Barbuda, Argentina, Armenia, Aruba, Australia, Austria, Azerbaijan, Bahamas, Bahrain, Bangladesh, Barbados, Belarus, Belgium, Belize, Bermuda, Bhutan, Bolivia, Bosnia and Herzegovina, Botswana, Brazil, Brunei, Bulgaria, Cambodia, Cameroon, Canada, Cape Verde, Cayman Islands, Chile, China, Colombia, Congo, Costa Rica, Cote d’Ivoire, Croatia, Curacao, Cyprus, Czechia, Denmark, Djibouti, Dominica, Dominican Republic, Ecuador, Egypt, El Salvador, England, Equatorial Guinea, Estonia, Eswatini, Ethiopia, Faeroe Islands, Falkland Islands, Fiji, Finland, France, Gabon, Gambia, Georgia, Germany, Ghana, Gibraltar, Greece, Greenland, Grenada, Guatemala, Guernsey, Guinea, Guyana, Honduras, Hong Kong, Hungary, Iceland, India, Indonesia, Iran, Iraq, Ireland, Isle of Man, Israel, Italy, Jamaica, Japan, Jersey, Jordan, Kazakhstan, Kenya, Kosovo, Kuwait, Kyrgyzstan, Laos, Latvia, Lebanon, Lesotho, Libya, Liechtenstein, Lithuania, Luxembourg, Macao, Malawi, Malaysia, Maldives, Mali, Malta, Mauritania, Mauritius, Mexico, Moldova, Monaco, Mongolia, Montenegro, Montserrat, Morocco, Mozambique, Myanmar, Namibia, Nauru, Nepal, Netherlands, New Zealand, Nicaragua, Niger, Nigeria, North Macedonia, Northern Cyprus, Northern Ireland, Norway, Oman, Pakistan, Palestine, Panama, Papua New Guinea, Paraguay, Peru, Philippines, Poland, Portugal, Qatar, Romania, Russia, Rwanda, Saint Helena, Saint Kitts and Nevis, Saint Lucia, Saint Vincent and the Grenadines San Marino, Sao Tome and Principe, Saudi Arabia, Scotland, Senegal, Serbia, Seychelles, Sierra Leone, Singapore, Slovakia, Slovenia, Solomon Islands, Somalia, South Africa, South Korea, South Sudan, Spain, Sri Lanka, Sudan, Suriname, Sweden, Switzerland, Syria, Taiwan, Thailand, Timor, Togo, Tonga, Trinidad and Tobago, Tunisia, Turkey, Turks and Caicos Islands, Uganda, Ukraine, United Arab Emirates, United Kingdom, United States, Uruguay, Uzbekistan, Venezuela, Vietnam, Wales, Zambia, and Zimbabwe.

## Data Availability Statement

The original contributions presented in the study are included in the article/Supplementary Material, further inquiries can be directed to the corresponding author.

## Author Contributions

KF and AQ performed data acquisition and analysis. KF, BA, and AQ performed the results interpretation. All authors wrote the manuscript, contributed to the article, and approved the submitted version.

## Conflict of Interest

The authors declare that the research was conducted in the absence of any commercial or financial relationships that could be construed as a potential conflict of interest.
