# Supplementary material for: Correlation Between SARS-Cov-2 Vaccination, COVID-19 Incidence and Mortality: Tracking the Effect of Vaccination on Population Protection in Real Time
Source: Front Genet. 2021 Jun 2;12:679485. doi: 10.3389/fgene.2021.679485 (PMC8206786; doi:10.3389/fgene.2021.679485)
Supplement: Supplementary file 1 [file Table_1.docx]

Supplementary Table 1. Correlation between the numbers of vaccines against the number of applied tests in the globe (02.02.201).

| Countries | Tests | |
| --- | --- | --- |
|  | R coefficient | P value |
| Albania | 0.41565826 | 0.017984 |
| Algeria | NA | NA |
| Andorra | NA | NA |
| Argentina | -0.09122682 | 0.533019 |
| Austria | 0.281978335 | 0.086513 |
| Bahrain | 0.035942969 | 0.799829 |
| Bangladesh | 0.090961786 | 0.665434 |
| Barbados | NA | NA |
| Belgium | 0.181974118 | 0.187849 |
| Bolivia | -0.28602878 | 0.32151 |
| Brazil | NA | NA |
| Bulgaria | 0.355041628 | 0.015926 |
| Cambodia | NA | NA |
| Canada | -0.42105263 | 0.073952 |
| Chile | -0.01720212 | 0.898018 |
| China | NA | NA |
| Colombia | -0.5 | 0.45 |
| Costa Rica | NA | NA |
| Croatia | 0.051806669 | 0.750881 |
| Cyprus | 0.654431666 | 1.08E-06 |
| Czechia | 0.046753247 | 0.731583 |
| Denmark | 0.112885019 | 0.419827 |
| Ecuador | 0.075753495 | 0.707258 |
| Estonia | 0.168329155 | 0.219268 |
| Finland | 0.384928727 | 0.005285 |
| France | 0.164734816 | 0.238492 |
| Germany | NA | NA |
| Greece | 0.443476859 | 0.000986 |
| Hungary | 0.299134199 | 0.026897 |
| Iceland | -0.0569798 | 0.691255 |
| India | -0.2843029 | 0.121126 |
| Indonesia | -0.3559171 | 0.074337 |
| Iran | NA | NA |
| Ireland | -0.131258 | 0.368661 |
| Israel | -0.2854396 | 0.022557 |
| Italy | 0.2423103 | 0.07213 |
| Kuwait | 0.3091607 | 0.030655 |
| Latvia | -0.1673033 | 0.342869 |
| Lebanon | NA | NA |
| Liechtenstein | NA | NA |
| Lithuania | -0.3831904 | 0.00448 |
| Luxembourg | 0.2729975 | 0.055089 |
| Maldives | 0.5857843 | 0.015227 |
| Malta | -1 | 1 |
| Mauritius | NA | NA |
| Mexico | 0.2391793 | 0.070571 |
| Morocco | -0.4039113 | 0.077365 |
| Myanmar | -0.8571429 | 0.02381 |
| Nepal | 0.1485221 | 0.70294 |
| Netherlands | NA | NA |
| Norway | -0.2450748 | 0.079926 |
| Oman | NA | NA |
| Pakistan | 0.0183494 | 0.957296 |
| Panama | 0.5736104 | 0.00176 |
| Peru | NA | NA |
| Poland | -0.0393586 | 0.775417 |
| Portugal | 0.0309058 | NA |
| Qatar | 0.4508885 | 0.000551 |
| Romania | 0.0282258 | 0.87818 |
| Russia | -0.2855852 | 0.046687 |
| Saudi Arabia | -0.2013384 | NA |
| Serbia | -0.107996 | 0.524625 |
| Seychelles | NA | NA |
| Singapore | NA | NA |
| Slovakia | 0.5344588 | 0.000138 |
| Slovenia | 0.1360775 | 0.321873 |
| South Africa | -0.9746794 | 0.004818 |
| Spain | NA | NA |
| Sri Lanka | -0.4565217 | 0.026086 |
| Sweden | -0.5758887 | 1.49E-05 |
| Switzerland | -0.1373276 | 0.542244 |
| Turkey | -0.357085 | 0.026227 |
| United Arab Emirates | -0.039837 | 0.790347 |
| United Kingdom | 0.5995848 | 8.26E-08 |
| United States | -0.2157738 | 0.089462 |

NA = Correlation could not be performed because of low number of observations
